# The Role, Challenges, and Employment Characteristics of Disability Resource Professionals in Medical Education: A National Study

**DOI:** 10.1177/23821205251344771

**Published:** 2025-05-30

**Authors:** Erin Broskowski, Sarah E. Triano, Kara James, Rylee Betchkal, Karyn LaTurner Echols, Suzanne Hawks, Ochanya Ogah, Mytien Nguyen, Lisa M. Meeks

**Affiliations:** 1 Disability Resource Center, 12247 University of Illinois Chicago College of Medicine, Chicago, IL, USA; 2 Division of Student Affairs, 150362 Geisinger Commonwealth School of Medicine, Scranton, PA, USA; 3 Academic Support and Well-Being, 12270 Mayo Clinic College of Medicine and Science, Rochester, MN, USA; 4 Counseling and Clinical Psychology, 5930 Teachers College at Columbia University, New York, NY, USA; 5 Student Affairs, 149991 Rocky Vista University College of Osteopathic Medicine, Ivins, UT, USA; 6 8676 Wake Forest University, Winston-Salem, NC, USA; 7 Department of Internal Medicine, 12224 UCSF School of Medicine, San Francisco, CA, USA; 8 School of Medicine, 12228 Yale University School of Medicine, New Haven, CT, USA; 9 Department of Medical Education, 12247 University of Illinois College of Medicine, Chicago, IL, USA

**Keywords:** disability resource professionals (DRPs), medical education, disability, reasonable accommodation, faculty attitudes

## Abstract

**Background:**

Disability disclosure and accommodation requests among US medical students have increased significantly, yet gaps in support persist. Disability resource professionals (DRPs) are one essential support in bridging these gaps, but only 9% of medical schools employ a dedicated DRP, leaving institutions potentially underprepared to address the needs of students with disabilities.

**Objective:**

This study explored the roles, qualifications, and challenges of DRPs in US medical schools to identify barriers to job performance and inform strategies for strengthening and supporting this role in medical education.

**Methods:**

An exploratory, cross-sectional survey was conducted in July to August 2023. A 27-question online survey was distributed through convenience and snowball strategy via social media and listservs. Questions addressed institutional structures, the size of DRP student caseloads, job satisfaction, mentorship, and barriers to managing caseloads. Quantitative data were analyzed using descriptive statistics and χ^2^ tests, qualitative responses were thematically analyzed. The University of Michigan IRB approved this study.

**Results:**

Seventy-nine DRPs from 72 US MD and 7 DO programs participated. Most respondents reported excessive workloads, with 30% managing caseloads exceeding 100 students. Less than half of DRPs (45.6%) indicated that their workload was manageable. Inadequate professional development, lack of mentorship, and low salary satisfaction emerged as common challenges. Respondents also reported institutional barriers, including stigma, faculty resistance to accommodations, and the complexity of coordinating disability accommodations across didactic, clinical, and testing environments.

**Discussion:**

DRPs are critical to fostering accessible and inclusive medical education, yet systemic barriers undermine their effectiveness and place institutions at risk of increased student attrition, legal liability, and resource strain. Addressing these challenges requires investments in manageable caseloads, professional development for DRPs, faculty training, and institutional prioritization of the DRP role.

**Conclusions:**

Systemic investments in staffing, training, and institutional culture are necessary to support DRPs and the growing population of US medical students with disabilities (MSWDs).

## Background

Disability disclosure to US medical schools and subsequent requests for accommodations have risen sharply since 2015.^1–2^ This growth is reflected in self-reported measures of disability, including on the Association of American Medical Colleges’ (AAMC) Year Two Questionnaire (Y2Q), where the percentage of medical students disclosing a disability increased from 10.8% in 2020^2^ to 17.6% in 2024.^3^ Despite this rise, fewer than half (43.4%) of medical students who self-disclosed a disability reported receiving accommodations from their medical school in 2024, highlighting a critical and persistent gap in support for medical students with disabilities (MSWDs).^3^ This gap is often driven by concerns about bias, stigma, and misconceptions about performance.^4–10^ These individual-level barriers are exacerbated by structural barriers such as burdensome processes, conflict of interest, and limited institutional understanding of clinical accommodations.^4,10–16^

It has been suggested that specialized disability resource professionals (DRPs) play a critical role in bridging the gap between disclosure and accommodations.^4,16,17^ Both the AAMC and the American Medical Association (AMA) propose the necessity of this role in their respective reports on disability inclusion.^4,18^ This is strengthened by calls in the literature emphasizing the need for disability specialization to address medical students’ unique needs.^4,10,11,16–18^ Despite strong endorsements from the AAMC and the AMA, fewer than one in ten U.S. medical schools currently employ a DRP with specialized training in medical education,^11^ highlighting a significant gap in the infrastructure necessary to support disability inclusion in medical education.

To effectively address this unmet need, a thorough understanding of the DRP role, as well as DRPs' qualifications, experiences, and challenges, is needed; however, no study to date has reviewed the credentials and experiences of DRPs currently serving medical education. To address this gap, we conducted an exploratory survey of US medical school DRPs to examine their roles, preparation, and experiences, including titles, caseloads, salaries, education, mentorship, job satisfaction, and challenges to managing their caseload. By understanding the experiences of DRPs, this study provides actionable insights to guide efforts toward standardizing and strengthening the critical role of DRPs in fostering a more inclusive medical education environment.

## Methods

An exploratory, cross-sectional survey of medical school DRPs was conducted in July through August 2023. Survey respondents were recruited by convenience and snowball sampling strategies via LinkedIn and Twitter, as well as 3 listservs focused on disability access in medical education. Posts were made across all platforms three times at one-week intervals. A researcher and 2 DRPs (LM, EB, ST) designed the SurveyMonkey^®^^19^ online open survey, which included 27 questions: 26 multiple-choice and 1 open-ended question. The usability and technical functionality of the electronic questionnaire were tested by 4 additional DRPs before fielding the survey. US DRPs working in MD and DO-granting medical schools were eligible to complete the survey. The survey addressed institutional characteristics, DRP roles (e.g., caseload size, job duties, and resources), mentorship, departmental support, and professional development needs. Participants were informed of the study's goals and assured of confidentiality, with data reported in aggregate. Missing data for one question (satisfaction with mentor) are reported in the tables. No personally identifying information was collected, and no incentives were offered to respondents. The reporting of this study conforms to the Checklist for Reporting of Survey Studies.^20^

### Statistical Analysis

Quantitative responses were analyzed using descriptive statistics. Chi-square tests assessed associations between salary satisfaction, mentorship, school type, and resource structure. Differences in caseload satisfaction were examined using a Kruskal-Wallis test. Analyses were conducted in Stata version 18.0 (StataCorp).^21^

Open-ended responses to “What are the 3 biggest challenges when trying to manage your caseload?” were analyzed using qualitative coding. Two researchers (EB and ST) independently reviewed and coded a subset of responses to establish reliability. Themes emerged through an inductive approach, with codes systematically refined and grouped into broader categories. An iterative process ensured consistency and accuracy in capturing key barriers. The University of Michigan IRB exempted this study.

## Results

A total of 79 respondents participated in the survey, representing 72 allopathic (MD) and 7 osteopathic (DO) schools across the US. This corresponds to 47% of MD programs and 16% of DO programs. Of the respondents, 38 (48.1%) reported serving as a DRP on a health-science campus (encompassing all health professions and biomedical programs, including a medical school), 5 (6.3%) served at stand-alone medical schools, and 9 (11.4%) worked on main campuses supporting both undergraduate and professional students. Additionally, 27 (34.2%) respondents reported holding non-DRP roles while performing DRP responsibilities.

Respondents reported varying education backgrounds, ranging from bachelor's degrees to advanced degrees, including EdD, PhD, JD, and MD/DO, with the most commonly reported credential being a master's degree (49.4%). Reported DRP salaries varied from $50,000 to over $120,000, with the most frequently reported range falling between $50,000 and $70,000. Thirty percent of respondents reported managing caseloads exceeding 100 students. Regarding mentorship, 67.1% of respondents indicated they did not have a mentor in medical education, and among those with mentors, 58.2% reported dissatisfaction. Fifty-five percent of respondents reported feeling neutral or dissatisfied with their salary. When asked about caseload manageability, 45.6% indicated that their caseload was manageable (Table 1).

**Table 1.** Demographic and Employment Characteristics of Medical Education DRPs (N = 79).

Category	n (%)
**School type**	
US Allopathic (MD-granting)	72 (91.1%)
US Osteopathic (DO-granting)	7 (8.9%)
**Structure of disability resource office**	
Health-science campus (all health professions/biomedical programs, incl. medical school)	38 (48.1%)
Stand-alone medical school	5 (6.3%)
Main campus (undergraduate and graduate students, incl. medical students)	9 (11.4%)
Non-DRP role completing DRP tasks	27 (34.2%)
**Degree-level specialized DRP**	
Bachelor's	29 (36.7%)
Master's degree	39 (49.4%)
Doctorate (EdD, PhD)	8 (10.1%)
Terminal professional degree (e.g., law degree (JD); medical degree (MD/DO))	3 (3.8%)
**Salary range DRP (US dollars)**	
50-70K	26 (32.9%)
70-90K	25 (31.6%)
90-120K	17 (21.5%)
>120K	11 (13.9%)
**Medical student caseload for specialized DRP**	
50 or under	27 (34.2%)
50-100	22 (27.8%)
101-150	12 (15.2%)
150-280	18 (22.8%)
**Medical school mentor**	
Yes	26 (32.9%)
No	53 (67.1%)

Chi-square analysis between satisfaction with salary and access to mentors (*P* = .942), structure of disability resource (*P* = .415), and school type (*P* = .520) demonstrated no significant association between satisfaction with salary and these factors. Furthermore, there was no significant association between overall or medicine-related caseload with reported satisfaction with salary reported by DRPs (mean caseload among those who did not report satisfaction with salary: 476[79] vs mean caseload among those who reported satisfaction with salary: 538[163], *P* = .20; mean caseload among those who did not report satisfaction with salary: 78[11] vs mean caseload among those who reported satisfaction with salary: 84[8], *P* = .15). The majority of DRPs reported that their caseloads were not manageable (25, 31.6%) or were only manageable at times (18, 22.8%, Table 2).

**Table 2.** DRP Satisfaction With Salary and Mentorship and Ability to Manage Caseload.

**Satisfaction with salary**	
Dissatisfied	43 (54.4%)
Satisfied	36 (45.6%)
**Caseload manageable**	
Yes	36 (45.6%)
No	25 (31.6%)
At times	18 (22.8%)
**Satisfaction with mentor**	
Yes	15 (19.0%)
No	46 (58.2%)
Missing	18 (22.8%)

### Thematic Barriers to Job Performance for DRPs

Qualitative analysis of free responses to “What are the 3 biggest challenges when trying to manage your caseload?” highlighted significant challenges faced by DRPs in their professional roles. Understaffing and workload were the most frequently reported challenges, with respondents describing the significant time required to support students, particularly for high-stakes exams, and to educate faculty on accommodations. Insufficient staffing and unmanageable caseloads, with some DRPs supporting hundreds of students, further limited their ability to provide individualized services. Even when caseloads were described as manageable, competing responsibilities outside disability support created additional barriers. As one respondent noted, they “[did not have enough] time for necessary high-touch conversations… and administrative work,” illustrating the strain caused by balancing multiple tasks (Table 3).

**Table 3.** Thematic Barriers to Job Performance for DRP.

Main theme	Codes	Example quotes
Perception of disability	Attitudinal Barriers and Culture	• One individual wrote, “*Faculty are still in the mindset that students shouldn’t have accommodations in these professions.”* Many remarked on the reluctance of the administration and/or faculty to recognize the need for accommodations and the ongoing efforts required to educate and advocate against ableism. • Additionally, many DRPs report encountering “*students who register for accommodation late after they struggle due to the stigma.”* This makes it difficult for DRPs to determine and implement accommodations proactively. • One DRP noted that issues related to the perception of disability at their institution are a main focus of their work. “*Change management has absorbed a majority of my time - this includes the change for faculty/staff to promote a new understanding of equal access and for the students who are transitioning into graduate medical/allied health programs.”*
Complexity of DRP role and medical school infrastructure	Complexity, Poor Infrastructure, Licensing, Accommodation Process, and Administrative Burden	• Many DRPs reported challenges related to the complexity of needs and accommodations. One DRP response illustrates this by the following statement: “*[A significant challenge is] trying to keep up with all of the program directors, unit directors, education managers, program coordinators, time and lack of education about requirements and our office's needs/role, and complexity of accommodation requests, coordinating with faculty, scheduling and performing check-ins.”* • Accommodations must be customized to meet each student's needs while aligning with the technical standards and requirements of the specific educational program, which will differ across schools and degrees. One respondent listed “*Clinical accommodations and varying thresholds of reasonable, technical standards and how they come into play during the review process”* as challenges. • One DRP commented on the struggle to have a strong command of “*the complexities of undergraduate and postgraduate [sic] (residency) medicine programs and navigate relationships with dozens of stakeholders. Coordinating appropriate accommodations for both the lab and clinical environments.”* • Some respondents indicated the need for support with administrative duties to free up more time for specialized tasks. One respondent stated they have *“no administrative support (making appointments, updating database–things that do not need to be done by me),”* and another stated, “*As health science accommodations can often take a lot of collaboration to determine implementation, the additional administrative follow-up processes can be difficult.”* • One area where many of these challenges intersect is supporting students who apply for accommodations on high-stakes exams. Respondents remarked on the extensive time and administrative work required to effectively support students through this arduous and daunting process.
Professional development	Lack of Specialization, Training, and Education	• Many DRPs reported the difficulty of gaining the specialized knowledge necessary to operate within a medical school setting. • One DRP expressed frustration with the learning curve for such a highly specialized role; they said, “*This was a role that I am unfamiliar with. It was difficult* *…* *learning various technical standards of various departments.”* • Another expressed how providing specialized accommodation assistance was even more complicated when they operated within an office that only supported generalists: “*Trying to become a specialist in a generalist model is hard to do.”* • Another DRP noted that a lack of opportunities and support to gain this specialized knowledge prevented them from optimal performance in their work: *“[a significant challenge is] lack of education about requirements and our office's needs/ role.”*

Attitudinal barriers surrounding the perception of disability also emerged as a key theme. Respondents reported faculty resistance to accommodations and noted that stigma often led to late student disclosures, resulting in reactive, rather than proactive, processes. One respondent highlighted this issue, stating, “Faculty are still in the mindset that students shouldn’t have accommodations in these professions,” reflecting entrenched skepticism within the institutional culture.

The complexity of the DRP role and inadequate infrastructure added further challenges. Respondents highlighted the time and expertise required to coordinate accommodations across clinical, didactic, and high-stakes testing environments while navigating institutional processes. This complexity is compounded by the need to interact with numerous stakeholders and tailor accommodations to meet technical standards. As one DRP explained, “[A significant challenge is] coordinating appropriate accommodations for both the lab and clinical environments,” underscoring the effort required to meet diverse programmatic needs, particularly for dual-degree program students.

Finally, the lack of professional development opportunities, standardized training pathways, and resources left many DRPs without the specialized knowledge necessary to address the unique demands of medical education. One respondent expressed frustration with this gap, stating, “Trying to become a specialist in a generalist model is hard to do,” highlighting the difficulty of acquiring expertise in an unsupported environment.

## Discussion

This study provides critical insights into the employment landscape, challenges, and institutional consequences of barriers experienced by DRPs in US medical education. While DRPs are essential for ensuring compliance with disability legislation and equitable access for MSWDs, understaffing, excessive workloads, attitudinal barriers, and role complexity place institutions at significant risk across 4 critical areas: cost, attrition, climate, and personnel.

Attrition, financial strain, and personnel challenges are significant risks for institutions lacking robust DRP support. Without sufficient expertise, MSWDs denied accommodations may experience delays in academic progress,^6,22,23^ underperformances on critical exams like the United States Medical Licensing Examination (USMLE) Step 1,^23^ and an increased likelihood of remediation or withdrawal.^23^ These challenges can place additional strain on institutional resources, including faculty, advisors, and student affairs teams,^23^ and may harm MSWDs' graduation rates and long-term outcomes.

Failure to provide adequate accommodations may also expose institutions to legal liability under disability legislation, including the Americans with Disabilities Act^24^ and Section 504 of the Rehabilitation Act.^25^ Non-compliance could result in costly lawsuits, settlements, and increased staff involvement, further straining institutional resources. Additionally, institutions risk losing experienced DRPs due to overwhelming workloads, lack of administrative support, and insufficient professional development opportunities. Burnout and turnover among DRPs may disrupt institutional operations and further delay critical support for MSWDs. Given the specialized expertise required for this role, replacing skilled DRPs can be challenging and resource-intensive, leaving institutions vulnerable to gaps in support and compliance.

Attitudinal barriers, including stigma and skepticism toward accommodations, undermine the institutional climate by discouraging disability disclosure and eroding trust. A lack of visible investment in disability resources, such as DRPs, may contribute to poor student engagement with services, increased attrition, and a diminished sense of inclusion and belonging.^6^

The growing enrollment of MSWDs marks a positive shift toward a more diversified workforce that better reflects the patients they will serve.^26^ To fully realize this progress, institutions must prioritize the development and sustainability of the DRP role. By addressing barriers such as staffing, professional development, and role complexity, institutions can ensure that DRPs are equipped to provide effective support, meet legal obligations, and foster inclusive learning environments that benefit all students.

Investing in DRPs demonstrates a strategic commitment to student success and long-term resource sustainability. As the number of MSWDs continues to grow, institutions must proactively strengthen the infrastructure needed to support this progress, ensuring that all learners have the opportunity to thrive.

## Limitations

This study's use of convenience and snowball sampling may introduce selection bias, potentially overrepresenting DRPs who are inclined to respond. While generalizability is limited, the sample includes representation from both MD and DO programs. Notably, the consistency of qualitative responses across institutions suggests shared challenges and experiences, offering valuable insights into the field.

## Recommendations

To mitigate the risks identified, institutions must implement focused strategies to better support DRPs and, by extension, MSWDs. First, institutions should establish manageable caseload limits to ensure DRPs can provide both the depth and breadth of support necessary for MSWDs.^27^ This aligns with recommendations in the literature and promotes higher-quality, individualized services.^4,27^

Second, investing in professional development opportunities is critical. Partnerships with organizations such as the AAMC,^28^ Association on Higher Education and Disability (AHEAD),^29^ and DocsWithDisabilities Initiative^30^ offer low- or no-cost resources for DRPs to deepen their expertise. However, institutions must prioritize releasing time for DRPs to attend these events and training opportunities. Supporting DRPs in their ongoing education enhances their effectiveness and signals an institutional commitment to disability inclusion.

Third, institutions should prioritize faculty anti-ableism training to attenuate the need for individualized instruction from DRPs. Structured faculty training on accommodations (both didactic and clinical), disability law, technical standards, and the benefits of training MSWDs may reduce DRP burden while fostering inclusion. Quarterly DRP updates for staff and leadership can further encourage proactive, institution-wide approaches.^31^

To elevate the role of the DRP, institutions must position these professionals as valued and respected partners in medical education. Leadership should set an example by visibly supporting DRPs as critical members of the educational team. Retention efforts must also be prioritized; DRPs should be compensated fairly, treated well, and given resources to excel in their roles. The institutional history and longitudinal relationships DRPs build with medical students and faculty are paramount to delivering effective and consistent support.

Finally, associations should consider developing a certificate program or series of courses to formally acknowledge advanced work and specialization in medical education DRP roles. Such recognition would validate the expertise required for these positions and provide DRPs with a clear pathway for career development and growth.

## Conclusion

The increase in the number of MSWDs signals progress toward a more diverse and representative healthcare workforce. The role of DRPs is critically important to support and retain these students in their path to physicianhood. Investing in DRPs has the potential to strengthen MSWDs outcomes, fulfill legal obligations, and advance equity in medical education.

## Supplemental Material

sj-pdf-1-mde-10.1177_23821205251344771 - Supplemental material for The Role, Challenges, and Employment Characteristics of Disability Resource Professionals in Medical Education: A National Study Supplemental material, sj-pdf-1-mde-10.1177_23821205251344771 for The Role, Challenges, and Employment Characteristics of Disability Resource Professionals in Medical Education: A National Study by Erin Broskowski, Sarah E. Triano, Kara James, Rylee Betchkal, Karyn LaTurner Echols, Suzanne Hawks, Ochanya Ogah, Mytien Nguyen and Lisa M. Meeks in Journal of Medical Education and Curricular Development

sj-docx-2-mde-10.1177_23821205251344771 - Supplemental material for The Role, Challenges, and Employment Characteristics of Disability Resource Professionals in Medical Education: A National Study Supplemental material, sj-docx-2-mde-10.1177_23821205251344771 for The Role, Challenges, and Employment Characteristics of Disability Resource Professionals in Medical Education: A National Study by Erin Broskowski, Sarah E. Triano, Kara James, Rylee Betchkal, Karyn LaTurner Echols, Suzanne Hawks, Ochanya Ogah, Mytien Nguyen and Lisa M. Meeks in Journal of Medical Education and Curricular Development
