# Supplementary material for: The Role, Challenges, and Employment Characteristics of Disability Resource Professionals in Medical Education: A National Study
Source: J Med Educ Curric Dev. 2025 May 30;12:23821205251344771. doi: 10.1177/23821205251344771 (PMC12125517; doi:10.1177/23821205251344771)
Supplement: sj-pdf-1-mde-10.1177_23821205251344771 - Supplemental material for The Role, Challenges, and Employment Characteristics of Disability Resource Professionals in Medical Education: A National Study [file sj-pdf-1-mde-10.1177_23821205251344771.pdf]

AIM DRP Career Survey 2023

DRP Career Survey for Medical School DRP

Thank you for engaging with our survey.

Our goal is to report our aggregate data in support of DRP's career development for DRP's working in Medical Education and to provide data that can be used to negotiate salary, title, and other career items.

This survey will end August 30th and a report will be generated and sent back to all AIM members.

All data will be kept confidential and reported out in aggregate only. Including your name and institution are optional.

Any questions can be forwarded to us at accessinmedicine@gmail.com

Our Best,
Lisa Meeks, PhD and Sarah Triano, MS, NCC, LPC

1. **Name (optional)**

2. **Institution (optional)**

* 3. **What type of medical school do you cover allopathic or osteopathic program?**

- ○ Allopathic (MD)
- ○ Osteopathic (DO)

4. **Please identify the geographical location of your institution.**

- ◯ **US: South** (Alabama, Arkansas, Delaware, Florida, Georgia, Kentucky, Louisiana, Maryland, Mississippi, North Carolina, Oklahoma, South Carolina, Tennessee, Texas, Virginia, West Virginia, District of Columbia)
- ◯ **US: Midwest** (Illinois, Indiana, Iowa, Kansas, Michigan, Minnesota, Missouri, Nebraska, North Dakota, Ohio, South Dakota, Wisconsin)
- ◯ **US: West** (Alaska, Arizona, California, Colorado, Hawaii, Idaho, Montana, Nevada, New Mexico, Oregon, Utah, Washington, Wyoming)
- ◯ **US: Northeast** (Connecticut, Maine, Massachusetts, New Hampshire, New Jersey, New York, Pennsylvania, Rhode Island, Vermont)
- ◯ **CA: Central Canada** (Ontario and Quebec)
- ◯ **CA: Prairie Provinces** (Manitoba, Saskatchewan and Alberta)
- ◯ **CA: West Coast** (British Columbia)
- ◯ **CA: Atlantic Region** (New Brunswick, Nova Scotia, Prince Edward Island, Newfoundland and Labrador)
- ◯ **CA: North** (Yukon, Northwest Territories and Nunavut)
- ◯ **Other** (please specify)

5. **Which option best describes the location of your school?**

- ◯ Rural
- ◯ Suburban
- ◯ Urban (under 500K population)
- ◯ Urban (between 500K to 1 Million population)
- ◯ Urban (Over 1 Million population)

6. **Please select the structure that best represents your role in disability services as a disability resource provider (DRP).**

- ◯ DRP for a stand alone medical school
- ◯ DRP for a health-science campus (e.g., all health professions and biomedical programs, including a medical school)
- ◯ I fill another role and being a DRP is part of my responsibility for a stand alone medical school
- ◯ I fill another role and being a DRP is part of my responsibility for a health science campus that includes a medical school
- ◯ Other (please specify)

7. **What title best represents your role in the office? If no option accurately depicts your role, please check "Other" and specify.**

- Access Coordinator
- Senior Access Specialist
- Assistant Director
- Associate Director
- Director
- Executive Director
- Dean over a division
- Assistive Technology Specialist
- Other (please specify)

8. **What proportion of your role is assigned to disability services? Please provide in percent FTE (0-100 FTE); for example, if you are 50% time your FTE is .50.**

9. **Do your duties include training faculty and liaising with faculty?**

- Yes
- No
- Other (please specify)

10. **Do your duties include supporting students applications for high-stakes licensing exams?**

- Yes
- No
- Other (please specify)

11. **Does your department have an administrative assistant?**

- Yes
- No

12. **What is your current case load of ALL students? This is your grand total, including both medical students, allied health science students, and undergraduate students.**

13. **What is your current caseload of medical students ONLY? This is a grand total-- it may be the same as your total number or it may not.**

14. **Do you feel your current caseload is manageable?**

- Yes
- No
- Other (please specify)

15. **What are the three biggest challenges when trying to manage your caseload?**

16. **Please select the option that best represents your salary for 100% FTE Equivalent. For example, if you are paid 100K for 80% time, your 100% FTE equivalent would be 120K.**

- Under 50K yearly for 100% Time
- Between 50-70K for 100% Time
- Between 70-90K for 100% Time
- Between 90-120K for 100% Time
- Between 120-140K for 100% Time
- Above 140K for 100% Time
- Other (please specify)

17. **How satisfied are you with your salary, respective to your responsibilities?**

- Very satisfied
- Satisfied
- Neither satisfied nor dissatisfied
- Dissatisfied
- Very dissatisfied

18. **Does your institution allocate funds in the budget for you to engage in professional development?**

- ◯ Yes
- ◯ No
- ◯ Other (please specify)

19. **If yes, what is your annual budget?**

20. **What resources do you use the most when seeking consultation on a DRP related case? Select all that apply.**

- ☐ Equal Access for Students With Disabilities: The Guide for Health Science and Professional Education
- ☐ A peer/personal network
- ☐ My supervisor
- ☐ AHEAD (including the list-serv)
- ☐ The Coalition (including the list-serv)
- ☐ DocsWithDisabilities (including the listserv)
- ☐ Our legal counsel
- ☐ A consulting agency
- ☐ Other (please specify)

21. **Do you have a mentor in Medical Education?**

- ◯ Yes
- ◯ No
- ◯ Other (please specify)

22. **How satisfied are you with the level of mentoring you receive?**

- ◯ Very satisfied
- ◯ Satisfied
- ◯ Dissatisfied
- ◯ Very dissatisfied

23. **Are you interested in advanced training for specializing in accessibility services in medical education?**

- ◯ Yes
- ◯ No

24. **Which of the following populations have you worked with? Check all that apply.**

- ☐ Wheelchair user with an intermediary (assistant) for clinical work
- ☐ Wheelchair user without an intermediary
- ☐ Deaf and hard of hearing students with ASL or Designated interpreter
- ☐ Deaf and hard of hearing student with captioning in clinic
- ☐ Hard of hearing student with captioning in clinic
- ☐ Hard of hearing student with captioning in coursework
- ☐ Student with learning disability who uses text-to-speech assistive technology in the clinic
- ☐ Student with chronic illness who requires flexibility during clinical experiences
- ☐ Student with psychological disability who requires flexibility during clinical experiences
- ☐ Student with low vision who requires accommodations in clinical settings (including on EHR)
- ☐ None of the above

25. **Which of the following populations/scenarios would you like to receive training on? Check all that apply.**

- ☐ Wheelchair user with an intermediary (assistant) for clinical work
- ☐ Wheelchair user without an intermediary
- ☐ Deaf and hard of hearing students with ASL or Designated interpreter
- ☐ Deaf and hard of hearing student with captioning in clinic
- ☐ Hard of hearing student with captioning in clinic
- ☐ Hard of hearing student with captioning in coursework
- ☐ Student with learning disability who uses text-to-speech assistive technology in clinic
- ☐ Student with chronic illness who requires flexibility during clinical experiences
- ☐ Student with psychological disability who requires flexibility during clinical experiences
- ☐ Student with low vision who requires accommodations in clinical settings (including on EHR)
- ☐ None of the above

26. **Would you like any additional information or services related to bolstering your career?**

27. Please select your educational degree.

- ○ Bachelors Degree
- ○ Masters Degree (in any field)
- ○ Doctorate (PhD)
- ○ Doctorate (EdD)
- ○ Law Degree (JD)
- ○ Medical Degree (DO/MD)
